# Insulin Substrate Receptor (IRS) proteins in normal and malignant hematopoiesis

**DOI:** 10.6061/clinics/2018/e566s

**Published:** 2018-10-03

**Authors:** João Agostinho Machado-Neto, Bruna Alves Fenerich, Ana Paula Nunes Rodrigues Alves, Jaqueline Cristina Fernandes, Renata Scopim-Ribeiro, Juan Luiz Coelho-Silva, Fabiola Traina

**Affiliations:** I Departamento de Medicina Interna, Faculdade de Medicina de Ribeirao Preto, Universidade de Sao Paulo, Ribeirao Preto, Sao Paulo, SP, BR; II Departamento de Farmacologia do Instituto de Ciencias Biomedicas da Universidade de Sao Paulo, Sao Paulo, SP, BR

**Keywords:** Insulin Receptor Substrate, Adaptor Protein, Signal Transduction, Hematopoiesis, Leukemia, Myeloproliferative Neoplasms

## Abstract

The insulin receptor substrate (IRS) proteins are a family of cytoplasmic proteins that integrate and coordinate the transmission of signals from the extracellular to the intracellular environment via transmembrane receptors, thus regulating cell growth, metabolism, survival and proliferation. The PI3K/AKT/mTOR and MAPK signaling pathways are the best-characterized downstream signaling pathways activated by IRS signaling (canonical pathways). However, novel signaling axes involving IRS proteins (noncanonical pathways) have recently been identified in solid tumor and hematologic neoplasm models. Insulin receptor substrate-1 (IRS1) and insulin receptor substrate-2 (IRS2) are the best-characterized IRS proteins in hematologic-related processes. IRS2 binds to important cellular receptors involved in normal hematopoiesis (EPOR, MPL and IGF1R). Moreover, the identification of IRS1/ABL1 and IRS2/JAK2^V617F^ interactions and their functional consequences has opened a new frontier for investigating the roles of the IRS protein family in malignant hematopoiesis. Insulin receptor substrate-4 (IRS4) is absent in normal hematopoietic tissues but may be expressed under abnormal conditions. Moreover, insulin receptor substrate-5 (DOK4) and insulin receptor substrate-6 (DOK5) are linked to lymphocyte regulation. An improved understanding of the signaling pathways mediated by IRS proteins in hematopoiesis-related processes, along with the increased development of agonists and antagonists of these signaling axes, may generate new therapeutic approaches for hematological diseases. The scope of this review is to recapitulate and review the evidence for the functions of IRS proteins in normal and malignant hematopoiesis.

## INTRODUCTION

The insulin receptor substrate (IRS) proteins are a family of cytoplasmic proteins composed of six members (IRS1-6) that act as adaptor proteins 1-6. IRS proteins integrate and coordinate multiple cellular processes by transducing signals from the extracellular to the intracellular environment via transmembrane receptors 1 and are the major molecules that mediate the response to insulin and insulin-like growth factor 1 (IGF1) stimulation 2,7. IRS proteins regulate numerous processes such as growth, metabolism, survival and proliferation, and they respond to various stimuli, including steroids, cytokines, hormones and integrins [reviewed in 8 and 9].

**Figure 1:**
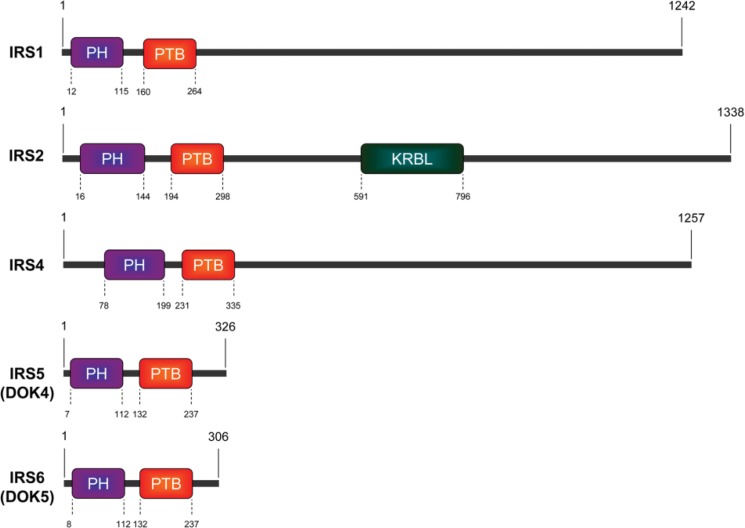
Schematic of human IRS protein structures. The pleckstrin homology (PH) domain, phosphotyrosine binding (PTB) domain and kinase regulatory loop binding (KRLB) domain are shown in the figure. Amino acid (aa) positions are indicated.

IRS1 was the first member of the IRS protein family to be identified and cloned 10. IRS2 was identified in Irs1-knockout mice as a phosphoprotein that responds to insulin stimulation 11. In humans, IRS3 is a pseudogene 12. The expression of IRS4 is restricted to the brain, kidney, thymus and liver 5. IRS5 and IRS6, also called docking protein-4 (DOK4) and docking protein-5 (DOK5), respectively, have high homology with other members of the IRS protein family in their N-terminal regions 6,13. The structures of the human IRS proteins are shown in Figure 1.

IRS proteins do not have kinase or other intrinsic enzymatic activity; however, they contribute to the organization of signaling complexes as adaptor proteins 2. IRS proteins have high levels of homology in the N-terminal regions, which contain two conserved domains that participate in receptor recruitment: the pleckstrin homology (PH) domain and the phosphotyrosine binding (PTB) domain. The PH domain participates in protein-protein interactions and facilitates recruitment by receptors and phospholipid proteins located in the plasma membrane 14-16. The PTB domain contains the tyrosine residues that interact with NPXY motifs on activated receptors 17,18. The activation of IRS proteins occurs after the phosphorylation of tyrosine residues in the C-terminal region, which contains more than twenty tyrosine sites. When phosphorylated, IRS proteins can bind to various Src homology (SH2) domain-containing proteins, including PI3K, GRB2, SHP2, resulting in the activation of multiple signaling pathways, especially the PI3K/AKT/mTOR and MAPK pathways 19-23.

PI3K-mediated signaling plays a critical role in many cellular biological events, including mitogenesis, motility, metabolism and survival 24. The C-terminal region of the IRS proteins contains several YMXM motifs, which bind to the SH2 domain of the PI3K p85 subunit when phosphorylated, with the consequent activation of AKT 25. PI3K was originally identified as a dimer composed of a catalytic subunit (p110) and a regulatory subunit (p85). The binding of phosphorylated proteins to the SH2 domain of the PI3K p85 subunit activates the associated catalytic domain. PI3K catalyzes the phosphorylation of phosphoinositides at the 3-position of the inositol ring, producing phosphatidylinositol 3,4,5-triphosphate (PI(3,4,5)*P*3), which in turn activates intracellular substrates such as AKT 26. The antiapoptotic effect of AKT is associated with the phosphorylation of its substrates, including BAD, caspase 9, NF-kB and the family of forkhead transcription factors 27. BAD phosphorylation prevents its interaction with BCL2 and BCL-XL, allowing its antiapoptotic action on the mitochondrial pathway 28.

**Figure 2:**
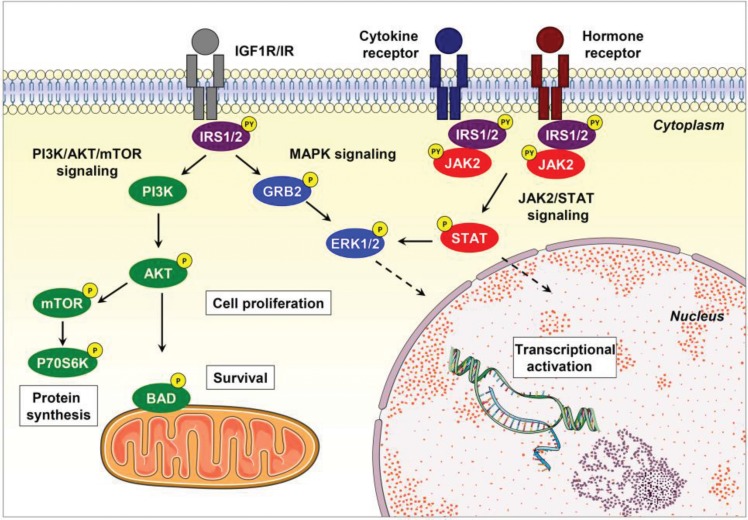
Canonical IRS signaling. IRS proteins are recruited via their PH/PTB domains and are phosphorylated on tyrosine residues by upstream tyrosine kinase receptors. Tyrosine phosphorylation of IRS proteins triggers the activation of PI3K/AKT/mTOR and MAPK signaling, thus regulating many biological processes, including cell proliferation, protein synthesis, survival and gene expression, in specific human tissues. IRS proteins may also be activated by cytokine and hormone receptors (*e.g.,* IL4, leptin, and angiotensin), which further induce JAK2 stimulation and IRS/JAK2 interaction, leading to the activation of STAT, PI3K/AKT/mTOR and MAPK signaling. Abbreviations: P, phosphorylation; PY, tyrosine phosphorylation. This figure was generated using Servier Medical Art (http://www.servier.com/Powerpoint-image-bank).

IRS proteins also bind to GRB2, leading to the activation of the MAPK cascade, which includes the ERK protein. The activation of the MAPK cascade is critical for cell differentiation and proliferation. In addition, IRS proteins may bind to other adapter proteins such as NCK, CRK, or the FYN kinase, also resulting in the activation of the MAPK cascade 20,29,30 (Figure 2).

Although IRS proteins have long been considered to exemplify typical cytosolic proteins, IRS1 may, under certain circumstances, be translocated to the nucleus, although the exact mechanism that promotes such translocation is not fully understood 31. Prisco et al. 32 noted that IRS1 contains native nuclear localization signals (NLSs), which may explain the translocation of IRS1 to the nucleus after IGF1/IGF1R activation 33. In addition, the presence of nuclear IRS1 in cells expressing human JC virus T-antigen, SV40 T-antigen, integrins, estrogen receptor α (ERα) and estrogen receptor β (ERβ) indicates that IRS1 can be translocated via association with other NLS-equipped proteins 31,32,34-36. However, the role of nuclear IRS proteins is still undetermined.

Deregulation of the IRS protein has been implicated in human diseases, especially diabetes and cancer [reviewed in 8,9 and 37]. Herein, we review and recapitulate the evidence for the roles of IRS proteins in normal and malignant hematopoiesis, exploring the clinical, biological and functional descriptions of the involvement of this protein family in the field of hematology.

### IRS signaling in normal hematopoiesis

Hematopoiesis is strictly regulated by cytokines and growth factors 38. Both IRS1 and IRS2 are expressed in a wide spectrum of cells and tissues 39. Unpublished data from our research group indicate that in human CD34^+^ bone marrow cells, *IRS2* is the predominant transcript, whereas in human CD3^+^ lymphocytes, *IRS1* is highly expressed. Irs2 expression is predominant in murine hematopoietic cells 3,39.

Machado-Neto et al. 40 reported increased levels of IRS2 mRNA, protein and phosphorylation in models of lineage-differentiated cell lines, including erythroid-, granulocytic- and megakaryocytic-differentiated cells 40. In CD34^+^ cells from normal donors, *IRS2* expression was increased upon erythroid differentiation 40. In granulocytic-differentiated HL-60 cells induced by dimethylsulfoxide, IGF1 induced an increase in IRS2 but not IRS1 protein expression and tyrosine phosphorylation as well as in PI3K recruitment 41. The genes encoding IRS2 and IGF1R were more highly expressed in plasma cells than in B cells, indicating that the IGF1R/IRS2 signaling pathway plays an important role in plasma cell differentiation and function 42. These data highlight the involvement of IRS2 in hematopoietic cell differentiation.

IRS2 can be activated via three relevant transmembrane receptors involved in hematopoiesis: IGF1R, EPOR, and TPOR (MPL) 41,43,44. The role of IGFI and its receptor in the regulation of hematopoietic cell development has been studied widely. Most such studies are related to the ability of IGF1 to stimulate myelopoiesis and erythropoiesis 45,46. However, in adult organisms, IGF1 does not seem to be required for normal or malignant hematopoietic cell development 47. Another study demonstrated that although neither IGF1 nor insulin is required during early erythropoiesis, both play a role in the final stages of erythroid maturation via the phosphorylation of IRS2 48.

Erythropoietin (EPO) is the major regulator of erythropoiesis 49. Upon EPO binding, the erythropoietin receptor (EPOR) undergoes conformational changes and associates with JAK2 50-52. JAK2 can activate its associated signaling pathways via two distinct mechanisms: (*I*) an EPOR tyrosine phosphorylation-independent mechanism involving ERK1/2 stimulation 53; and (*II*) via the phosphorylation of numerous tyrosine residues in the cytoplasmic tail of the EPOR that act as docking sites for SH2 domain-containing proteins 52,54,55. IRS2 but not IRS1 is expressed in several murine and human EPO-sensitive cell lines, including cells with erythroid and megakaryocytic features.

In UT-7 cells stimulated with EPO, IRS2 is rapidly phosphorylated on tyrosine residues. Following EPO-induced tyrosine phosphorylation, IRS2 associates with two proteins: PI3K and PI-3,4,5-trisphosphate 5-phosphatase (SHIP). Furthermore, phosphorylated IRS2 remains constitutively associated with the EPOR 43. Sathyanarayana et al. 55 demonstrated that IRS2 is regulated by EPO at the transcriptional level in primary murine erythroblasts. Furthermore, using phosphoproteomic analysis to evaluate the potential adaptor proteins involved in EPOR/JAK2 signaling, Verma et al. 56 observed that IRS2 was phosphorylated on tyrosine residues 653, 675, 742 and 823 in response to EPO.

Thrombopoietin (TPO) is the pivotal signal that regulates platelet production; TPO binds to the MPL receptor on hematopoietic stem cells and megakaryocytes 57. The TPO-mediated association between the MPL receptor and IRS2 was described by Miyakawa et al. 44, who reported that TPO activates the PI3K pathway in BaF3/MPL cells via a complex comprising the p85 subunit of PI3K, phosphorylated SHP2 and GAB2 or a complex comprising the p85 subunit of PI3K and IRS2 44.

IRS proteins can also be activated by the interleukins (ILs) involved in hematopoiesis. In lymphoid cell lines, T cells and NK human lymphocytes, IRS1 and IRS2 are phosphorylated on tyrosine sites upon stimulation by IL2, IL4, IL7 and IL15 58. IL9 promotes the tyrosine phosphorylation of IRS1 by JAK tyrosine kinases in a murine T cell line (TS1) 59. Although most studies linking IRS proteins and hematopoiesis focus on IRS1 and IRS2, the expression of IRS5 and IRS6 in human T cells was reported, and IRS5 was identified as a negative regulator of T lymphocyte activation 60,61. Using 32D cells, a cell line that neither expresses endogenous IRS1 nor responds to IL4 or insulin, Wang et al. 62 demonstrated that IRS1 is required for insulin- and IL4-stimulated mitogenesis in hematopoietic cells. In 32D cells, the expression of IRS1 via transfection restored sensitivity to IL4 and insulin and induced proliferation 62. A subsequent study by the same research group showed different results; the stimulation of overexpressed IGF1R by IGF1 and IL4 induced hematopoietic cell proliferation independent of IRS expression and activation 63. IRS4 was also identified to participate in insulin and IL4 signaling in 32D cells 64. In hematopoietic cells, the type I interferon receptor can activate IRS signaling 65; interferon-α (IFN-α) binding induces the rapid tyrosine phosphorylation of IRS1 and IRS2, leading to the association of phosphorylated IRS proteins with PI3K 65-67.

### IRS signaling in myeloid neoplasms

#### Chronic myeloid leukemia

Traina et al. 68 were the first to demonstrate the involvement of the IRS1 protein in BCR-ABL1 signal transduction in chronic myeloid leukemia (CML). In the K562 cell line, a BCR-ABL1-positive cell line derived from a patient with CML in blast crisis, the IRS1 protein was constitutively phosphorylated and associated with BCR-ABL1, and IRS1 phosphorylation was inhibited by imatinib treatment. Traina et al. also described the association between IRS1 and PI3K and the IRS1-associated PI3K activity in K562 cells. The associations between these proteins were inhibited by imatinib treatment, suggesting that PI3K activation by BCR-ABL1 involves binding to the phosphorylated IRS1 protein and depends on the tyrosine kinase activity of BCR-ABL1. In K562 cells treated with imatinib and immunoprecipitated with an anti-GRB2 antibody, the GRB2-associated phosphorylation of both BCR-ABL1 and IRS1 was significantly reduced, suggesting the formation of a BCR-ABL1/IRS1/GRB2/PI3K complex 68.

The functional involvement of IRS1 in the BCR-ABL1 signaling pathway was later demonstrated using lentivirus-mediated IRS1 silencing with short hairpin RNA (shRNA) in K562 cells 69. IRS1 inhibition reduced cell proliferation and clonal growth by arresting the cell cycle in the G0/G1 phase. IRS1 inhibition also decreased AKT, P70S6K and ERK phosphorylation, indicating the downregulation of the PI3K/AKT/mTOR and MAPK pathways. The inhibition of IRS1 did not modulate apoptosis; BCL2, BAX and BAD; protein expression; or BCR-ABL1 and CRKL phosphorylation. IRS1 silencing was not synergistic with imatinib treatment 69.

Zhao et al. 70 identified IRS1 and IRS2 as inhibitory targets of miR-570 and verified that miR-570 is downregulated in CML clinical samples and in the K562 and LAMA-84 CML cell lines. This study revealed that the overexpression of miR-570 suppressed cell proliferation, increased apoptosis, and reduced glucose metabolism, whereas the inhibition of miR-570 increased cell proliferation, reduced apoptosis, and increased glucose metabolism. Corroborating the findings by Machado-Neto et al. 69, Zhao et al. 70 verified that in K562 cells, IRS1 or IRS2 silencing via small interfering RNA (siRNA) reduced cell viability and increased sensitivity to nutrient deprivation.

#### Myeloproliferative neoplasms

Previous studies have described the involvement of IRS1 and IRS2 in the JAK2 signaling pathway in nonhematologic cells. IRS1 was found to be associated with and phosphorylated by JAK2 in COS-1 cells overexpressing both IRS1 and JAK2 59. JAK2 coimmunoprecipitated with IRS2 in rat aortic smooth muscle cells and *in vivo* models following angiotensin II stimulation 71-74 and in rat livers following leptin stimulation 75. Considering the previous findings in nonhematologic tissues, our research group identified a constitutive protein association between IRS2 and JAK2 in myeloproliferative neoplasm (MPN) models, which present constitutive JAK2 activation due to a V617F mutation. In the HEL JAK2^V617F^ cell line, but not in U937 JAK2 wild-type leukemia cell lines, IRS2 was constitutively phosphorylated and associated with JAK2. In HEL cells, lentivirus-mediated IRS2 silencing decreased STAT5 phosphorylation, reduced cell viability and increased apoptosis. NT157, a pharmacological inhibitor of IGF1R/IRS1-2, reduced cell viability in JAK2^V617F^ primary MPN samples but not in JAK2 wild-type samples 76. A recent study using targeted next-generation sequencing identified IRS2 mutations in 2 of 16 (12.5%) patients with triple-negative MPN, one with polycythemia vera and the other with essential thrombocythemia 77.

#### Acute myeloid leukemia

In acute myeloid leukemia (AML), somatic mutations, aberrant gene/protein expression levels and activating autocrine loops may promote growth factor and cytokine signaling activation as well as clonal expansion 78. The activation of several prosurvival pathways in AML is an essential element in the optimization of molecular targeted therapies, such as those targeting proteins involved in the protein kinase C, STAT, MAPK, PI3K/AKT/mTOR pathways 79. IGF1 signaling is implicated in self-renewal/pluripotency in hematopoietic stem cell contexts and supports cell growth/survival via the activation of downstream pathways in both normal and neoplastic settings 80. In the hematopoietic context, the role of the insulin and insulin-like growth factor axis in AML treatment refractoriness has been studied, but the specific functions of the IRS proteins are underexplored.

Accumulating evidence demonstrates the role of IGF1R signaling via the PI3K/AKT/mTOR cascade in AML. IGF1 and other cytokines have been described as important for AML cell growth 81, and the activation of the IGF1R signaling pathway has been detected in cells from AML patients and contributes to the survival and proliferation of these cells 82,83. An association between increased activation of the IGF1R axis and resistance to cytarabine has been reported in leukemia. Blocking the IGF1R in a cytarabine-resistant cell line inhibited cell growth and led to apoptosis 84, suggesting that IGF1R and its downstream signaling pathways may provide valuable novel targets to overcome chemotherapeutic resistance in AML.

Bertacchini et al. 85 demonstrated that pharmacological inhibitors of PI3K (LY294002) and AKT (AKTi 1/2) induced apoptosis but could not abrogate the phosphorylation of AKT at serine 473 and threonine 308 in a group of primary AML samples. Indeed, 70% of the AML samples tested showed an increase in AKT phosphorylation after long-term exposure to inhibitors; this increase was related to an upregulation of IRS1 expression and IR, IGF1R and PDGFR phosphorylation. Taken together, these results confirm that in AML primary cells, IRS1 participates in a mechanism of resistance to PI3K signaling inhibition. Moreover, 75% of the AML primary cells resistant to AKT inhibitors presented high IGF1R/IRS1 phosphorylation, and the combination of AKT inhibitors and the IGF1R inhibitor linsitinib potentiated PI3K/AKT/mTOR inhibition 85. Thus, combination therapy could be an effective strategy for breaking the adaptive circuits formed in leukemia cells that render these cells resistant to therapy. Consistent with this hypothesis, Tamburini et al. 83 noted that mTORC1 inhibition by RAD001 increased AKT activation in primary AML cells as a consequence of IRS2 upregulation via autocrine activation of IGF1/IGF1R signaling. Collectively, these results provide evidence that IGF1R signaling mediated by IRS1 and IRS2 is involved in chemotherapeutic resistance in AML.

Genetic lesions that affect *TP53*, such as mutations and aneuploidy, are recognized as markers of a very dismal prognosis for AML patients 86. Recently, Quintás-Cardama et al. 87 demonstrated that the p53 pathway is frequently disrupted in AML, not just via *TP53* mutations/deletions but also via a molecular background permissive to the transformation capability of p53. Via a proteomic approach, increased IRS1 phosphorylation at serine 1101 was identified as a biological marker of p53 pathway deregulation 87.

#### Myelodysplastic syndrome

Our research group reported that *IRS2* expression was lower in bone marrow samples from patients with myelodysplastic syndrome (MDS) than in bone marrow samples from healthy donors 40. These findings agree with those of a previous study that used a microarray analysis to show that the level of IRS2 is lower in bone marrow mononuclear cells from MDS patients than in cells from healthy donors 88. *IRS2* expression was lower in MDS patients with ≥5% bone marrow blasts than in MDS patients with <5% bone marrow blasts, and *IRS2* downregulation was associated with an increased severity of cytopenia 40. These findings suggest that IRS2 deficiency may be related to ineffective hematopoiesis.

### IRS signaling in lymphoid neoplasms

#### Acute lymphoblastic leukemia

Fernandes et al. 89 recently identified high levels of IRS1 protein expression in acute lymphoblastic leukemia (ALL) cell lines and observed that IRS1 and β-catenin were colocalized in the nucleus and cytoplasm of all the lymphoid leukemia cell lines studied. In the cytoplasm of normal peripheral blood mononuclear cells, both proteins were only weakly detected, suggesting a lower activation of the IRS1/β-catenin axis in healthy donors than in patients with ALL. Fernandes et al. 89 also reported high *IRS1* and *β-catenin* mRNA expression in a cohort of forty-five adult patients with ALL compared to normal hematopoietic cells from thirteen healthy donors, indicating that the IRS1/β-catenin signaling pathway probably contributes to the pathophysiology of ALL. In mouse embryo fibroblasts, Chen et al. 90 previously described IRS1, via IGF1R signaling, as a protein responsible for the nuclear translocation and activation of β-catenin.

In the childhood ALL cell lines CCRF-CEM (T cell acute lymphoblastic leukemia, T-ALL), NALM6 (B cell acute lymphoblastic leukemia, B-ALL) and REH (B-ALL), Leclerc et al. 91 demonstrated that AMPK activation induced growth inhibition and apoptosis via the downregulation of mTOR phosphorylation on serine 2448. Moreover, the IGF1R/IRS1 axis was important in determining the pro- or antiapoptotic response to AMPK activators, since AMPK activation induced a compensatory survival response. This mechanism was mediated in part by the AMPK-induced phosphorylation of IRS1 on serine 794, which in turn activated downstream oncogenic pathways 91. Therefore, selected combination therapies using IRS1 inhibitors could be a potential strategy for ALL therapy.

In a study using primary cells from adult patients with B-ALL, Juric et al. 92 identified, via computational analysis of the data obtained by a microarray analysis, a lower expression of IRS1 in BCR-ABL1-positive ALL than in BCR-ABL1-negative ALL. In BCR-ABL1-positive ALL, IRS1 expression negatively correlated with survival, independent of age and leukocyte count at diagnosis 92.

The multitarget tyrosine kinase inhibitor GZD824 exhibited an antitumor effect in pre-B-ALL by inhibiting both the SRC kinase and PI3K/AKT/mTOR pathways, and ALL cells with lower IRS1 expression were more sensitive to GZD824 treatment than those with higher IRS1 expression. Therefore, IRS1 expression could be used as a biomarker to predict GZD824 efficacy in pre-B-ALL 93.

T-ALL cases involving IRS4 have rarely been reported since Karrman et al. 94 first reported, in 2009, the t(X;7)(q22;q34) translocation in a patient with childhood T-ALL. These researchers identified *IRS4* as the translocated gene and observed IRS4 overexpression 94. Another case appearing years later and reported by Kang et al. 95 presented a simultaneous translocation of the *TCR α/δ* loci (14q11) with different partner loci (Xq22 and 12p13), and fluorescent in situ hybridization suggested the involvement of the *IRS4* gene. In 2011, Karrman et al. 96, intrigued by the rare cases of T-ALL involving IRS4, identified IRS4 mutations in 2 of 21 (9.5%) patients with T-ALL. IRS4 is believed to exert mitogenic and proliferative effects more similar to the effects of IRS1 than to those of IRS2 8,94.

#### Chronic lymphocytic leukemia

High IGF1R expression was identified in primary chronic lymphocytic leukemia (CLL) cells, suggesting the contribution of the IGF1R/IRS signaling pathway to disease pathology. Treatment with IGF1R inhibitors (AG1024, PPP) and IGF1R/IR inhibitor (OSI-906) reduced the viability and induced apoptosis in CLL cells *in vitro*, independent of the presence of protective stromal cells, and reduced tumor burden *in vivo*. Pharmacological or siRNA inhibition of the IGF1R was associated with a significant reduction in IRS1, PI3K, AKT and ERK phosphorylation. These data indicate that in CLL cells, IGF1R signaling activates the PI3K/AKT and MAPK pathways via IRS1 97.

#### Multiple myeloma

Li and colleagues 98 demonstrated that IGF1R and downstream signaling pathways play an important role in the development of a broad spectrum of plasma cell tumors via constitutive IRS2 tyrosine phosphorylation and PI3K recruitment. In human multiple myeloma cell lines, IGF1 induced proliferation and antiapoptotic effects via IRS1-dependent PI3K/AKT and MAPK activation, even in IL6-independent cell lines, indicating that the IGF1R/IRS1 axis plays an important role in the development and progression of this disease 99.

Shi et al. 100 observed that low concentrations of mTOR inhibitors stimulated the PI3K/AKT cascade in multiple myeloma. These drugs, in addition to preventing the phosphorylation of the downstream mTOR targets p70S6K and 4EBP1 and subsequent G1 arrest, prevent IRS1 serine phosphorylation (at an inhibitory site). Therefore, the prevention of IRS serine phosphorylation enhanced the activity of IGF1R/IRS1 signaling pathways and downstream targets, such as PI3K/AKT/mTOR, independent of PTEN mutational status 100. This mechanism can be particularly detrimental in multiple myeloma, because IGF1R/IRS1-induced AKT activation is a protumoral stimulus in multiple myeloma cells 99,101. Thus, additional studies will be necessary to decipher the best strategy for combining mTOR inhibitors with other therapeutic agents in multiple myeloma 100.

#### Hairy cell leukemia

Recently, Durham et al. 102 identified, by next generation sequencing and copy number analysis, a novel gain-of-function mutation in IRS1 that contributed to clinical resistance to vemurafenib (BRAF^V600E^ inhibitor) in 1 of 53 (2%) patients with classical hairy cell leukemia. Moreover, these researchers observed that mutated IRS1 activated PI3K/AKT signaling and phosphorylated ERK1/2, leading to the cytokine-independent growth of Ba/F3 cells *in vitro* 102.

### Perspectives

**Figure 3:**
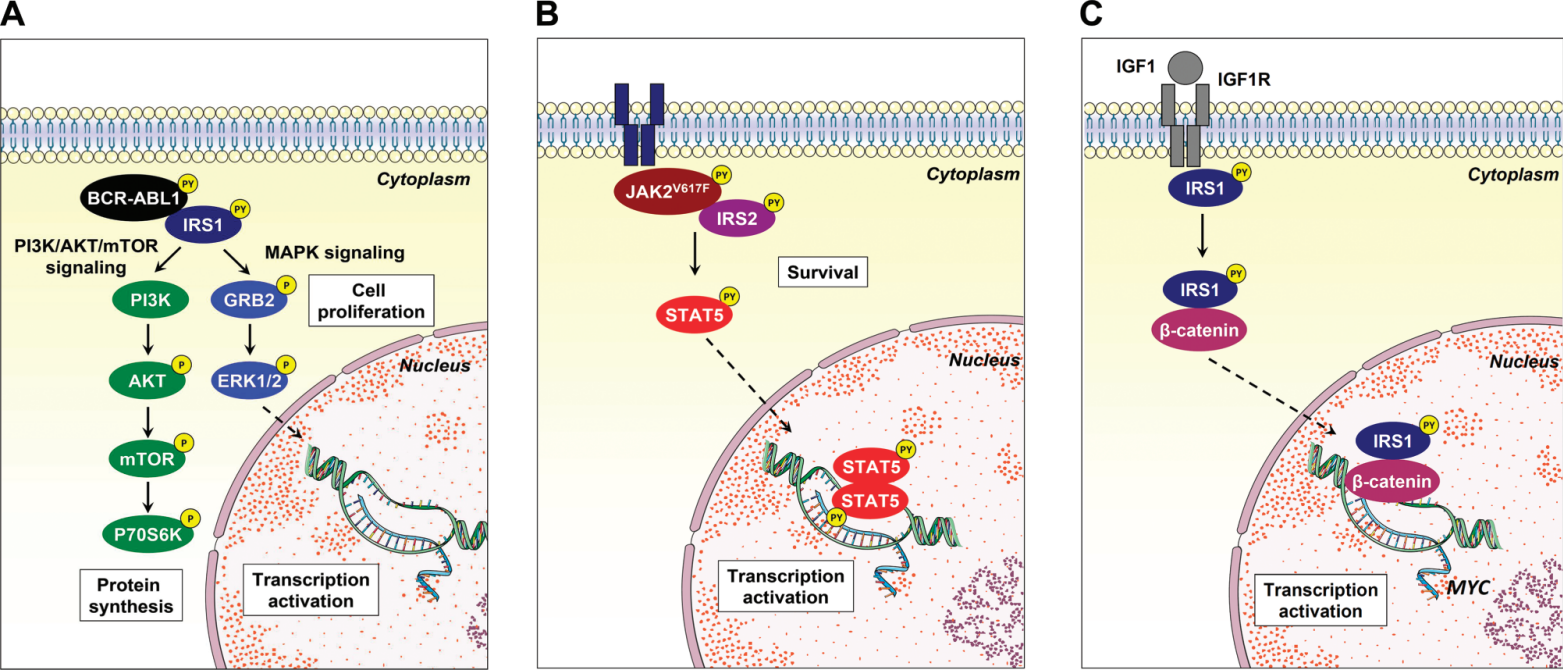
Noncanonical IRS1 signaling in hematological neoplasms. **(A)** IRS1 binds to and is activated by BCR-ABL1, inducing the activation of the PI3K/AKT/mTOR and MAPK signaling pathways, which contribute to cell proliferation. **(B)** IRS2 associates with JAK2 harboring the activating V617F mutation, which participates in STAT5 activation and cell survival. **(C)** Upon IGF1/IGF1R activation, IRS1 interacts with β-catenin, translocates to the nucleus and induces *MYC* expression in acute lymphoblastic leukemia cell lines. This figure was generated using Servier Medical Art (http://www.servier.com/Powerpoint-image-bank).

Studies using IRS protein (mainly IRS1, IRS2 and IRS4) knockout animals reveal that these animals are born alive but are smaller and present type II diabetes, reflecting the participation of IRS proteins in metabolic homeostasis 103-105. In oncology, IRS1 and IRS2 knockout mice as well as IRS1- and IRS2-overexpressing murine models were used to elucidate the function of these proteins in solid tumors, providing evidence of distinct and nonredundant functions for both proteins in cancer development and progression 106-108. However, despite the potential importance of IRS proteins in the signal transduction of hematopoietic-related growth factors and cytokines, as discussed herein, the function of this protein family in normal and malignant hematopoiesis remains poorly understood. Recently, a great effort has been undertaken to develop and identify compounds capable of inhibiting signaling mediated by the IR/IRS and IGF1R/IRS axes. Reuveni et al. 109 identified that NT157, a compound that binds to IGF1R and induces a conformational change leading to the dissociation of IRS1/2 from the receptor and to the degradation of IRS1/2 by the proteasome, presented antineoplastic effects in solid tumors 109-113. The cancer cell panel in the initial study included K562 (CML) and Karpas (lymphoma) cell lines; thus, the results suggested that NT157 may exert antileukemic effects 109. Similarly, GZD824, a multikinase inhibitor, downregulated IRS1 signaling and reduced cell viability and tumor burden both *in vitro* and in mice xenotransplanted with primary B-ALL cells 93. The participation of IRS1 and IRS2 in oncogenic pathways (namely, the BCR-ABL1 68,69, JAK2^V167F^ 76 and IRS1/β-catenin 89 pathways) described by our research group corroborates the participation of these proteins in the malignant phenotype of leukemias and suggests that these protein targets are druggable (Figure 3). Thus, a better understanding of the signaling pathway mediated by IRS proteins in hematopoietic-related processes, along with the increasing development of agonists and antagonists of this signaling axis, may generate new therapeutic approaches for hematological diseases.

In conclusion, the importance of IGF1R, EPOR and MPL signaling in cellular processes related to hematopoiesis has been recently consolidated; however, the mechanisms of intracellular regulation are continuously investigated. In this sense, the study of the participation of IRS proteins in hematopoietic processes still requires elucidation. The IRS proteins, particularly IRS1 and IRS2, play a relevant role in the signal transduction of membrane receptors and the neoplastic phenotype induced by oncogenes. A summary of IRS signaling pathway alterations in hematological neoplasms is presented in Table 1. Future studies on the involvement of IRS proteins are necessary to open new avenues and augment the understanding of the complex signaling mediating normal hematopoiesis and malignant transformation.

## ABBREVIATIONS

AKT, AKT serine/threonine kinase; AML, acute myeloid leukemia; AMPK, AMP-activated protein kinase; ALL, acute lymphoblastic leukemia; B-ALL, B cell acute lymphoblastic leukemia; BAD, BCL2-associated death promoter; BAX, Bcl-2-associated X protein; BCL2, B cell lymphoma 2; BCL-XL, B cell lymphoma-extra large; BCR-ABL1, breakpoint cluster region-Abelson 1; BRAF, B-Raf proto-oncogene, serine/threonine kinase; CD, cluster of differentiation; CLL, chronic lymphocytic leukemia; CML, chronic myeloid leukemia; CRK, CRK proto-oncogene, adaptor protein; CRKL, CRK-like proto-oncogene, adaptor protein; DOK, docking protein; EPO, erythropoietin; EPOR, erythropoietin receptor; ERα, estrogen receptor α; ERβ, estrogen receptor β; ERK, extracellular signal-regulated kinase; FYN, FYN proto-oncogene, Src family tyrosine kinase; GAB2, GRB2-associated-binding protein 2; GRB2, growth factor receptor-bound protein 2; IFN-α, interferon-α; IGF1, insulin-like growth factor 1; IGF1R, insulin-like growth factor 1 receptor; IL, interleukin; IR, insulin receptor; IRS, insulin receptor substrate; JAK2, Janus kinase 2; MAPK, mitogen-activated protein kinase; MDS, myelodysplastic syndrome; miR, Micro RNA; MPL, MPL proto-oncogene, thrombopoietin receptor; MPN, myeloproliferative neoplasms; mTOR, mammalian target of rapamycin; NCK, noncatalytic region of tyrosine kinase adaptor protein; NF-kB, nuclear factor-kappa B; NLS, nuclear localization signal; PDGFR, platelet-derived growth factor receptor; PH, pleckstrin homology; PI3K, phosphatidylinositol-4,5-bisphosphate 3-kinase; pre-B-ALL, B cell precursor acute lymphoblastic leukemia; PTB, phosphotyrosine binding; PTEN, phosphatase and tensin homolog; SH2, Src homology; SHIP, SH2-containing inositol phosphatase; SHP2, Src homology 2 domain-containing protein-tyrosine phosphatase 2; shRNA; short hairpin RNA; siRNA, small interfering RNA; SRC, SRC proto-oncogene, nonreceptor tyrosine kinase; STAT, signal transducer and activator of transcription; T-ALL, T cell acute lymphoblastic leukemia; TP53, tumor protein p53; TPO, thrombopoietin; TPOR, thrombopoietin receptor

## AUTHOR CONTRIBUTIONS

Machado-Neto JA, Fenerich BA, Rodrigues Alves APN, Fernandes JC, Scopim-Ribeiro R and Coelho-Silva JL participated in the preparation, completion and final approval of the manuscript. Traina F was the principal investigator and participated in the preparation, editing, completion and final approval of the manuscript. All authors read and approved the final version of the manuscript.

## Figures and Tables

**Table 1:** Alterations in the insulin receptor substrate (IRS) signaling pathway in hematological neoplasms.

Hematologic neoplasm	Sample/cell line	Notes	Main approaches	Publication
Chronic myeloid leukemia	K562	IRS1 is constitutively phosphorylated on tyrosine residues and associates with BCR-ABL1.	IP, WB	Traina et al.(68)
Chronic myeloid leukemia	K562	IRS1 silencing reduces cell proliferation and clonogenicity and inhibits mTOR/Akt and MAPK activation.	shRNA-lentiviral delivery	Machado-Neto et al. (69)
Chronic myeloid leukemia	K562 and LAMA-84	IRS1 and IRS2 silencing reduces cell viability and metabolism.	siRNA and transfection	Zhao et al. (70)
Philadelphia-negative myeloproliferative neoplasm	HEL, U937 and primary samples	IRS2 is associated with the JAK2^V617F^ mutation and induces survival in JAK2^V617F^-positive cells. NT157 reduces the viability of primary cells from MPN patients.	IP, WB and shRNA-lentiviral delivery	de Melo Campos et al. (76)
Acute myeloid leukemia	Primary samples	IRS1 mediates resistance to PI3K signaling inhibition.	WB	Bertacchini et al. (85)
Acute myeloid leukemia	Primary samples	IRS2 is upregulated by autocrine activation of IGF1/IGF1R signaling upon Akt/mTOR inhibitor treatment.	WB	Tamburini et al. (83)
Acute myeloid leukemia	Primary samples	IRS1 phosphorylation on serine 1101 is a biological marker of p53 pathway deregulation.	Proteomics and network analyses	Quintás-Cardama et al. (87)
Myelodysplastic syndrome	Primary samples	IRS2 is downregulated and is associated with an increased severity of cytopenia in MDS patients.	qPCR	Machado-Neto et al. (40)
Myelodysplastic syndrome	Primary samples	IRS2 is downregulated in bone marrow mononuclear cells from MDS patients compared with cells from healthy donors.	cDNA microarray	Bar et al. (88)
Acute lymphoblastic leukemia	Jurkat, MOLT4, Raji, Namalwa and primary samples	IRS1 is highly expressed in ALL cell lines and primary samples. Nuclear IRS1 associates with β-catenin and activates β-catenin signaling.	qPCR, WB and IP	Fernandes et al. (89)
Acute lymphoblastic leukemia	CCRF-CEM, NALM6 and REH	The activation of the IGF1R/IRS1 axis is a determinant of pro- or antiapoptotic responses to AMPK activators.	WB and cell viability assays	Leclerc et al. (91)
Acute lymphoblastic leukemia	Primary samples	IRS1 expression negatively correlates with survival, independent of age and leukocyte count at diagnosis.	cDNA Microarray	Juric et al. (92)
Acute lymphoblastic leukemia	Primary samples	IRS1 is a biomarker for the response to the multitarget tyrosine kinase inhibitor GZD824.	WB and cell viability assays	Ye et al. (93)
Acute lymphoblastic leukemia	Primary samples	IRS4 is translocated, overexpressed and mutated in ALL patients.	MC, FISH, WB and DNA sequencing	Karrman et al. (94)Kang et al. (95)Karrman et al. (96)
Chronic lymphocytic leukemia	Primary samples	IGF1R/IRS signaling is activated and promotes survival.	WB, cell viability assays and xenograft models	Yaktapour et al. (97)
Plasma cell neoplasms	Murine primary tumors	The activation of the IGF1R/IRS2/PI3K/p70S6K axis is important in the development of plasma cell tumors.	Transfection and allograft models	Li et al. (98)
Multiple myeloma	ANBL-6, Brown, Delta-47, OPM-2, 8226, KMM1, H929, and MM-144	Activation of the IGF1R/IRS1 axis leads to the inhibition of apoptosis and the induction of cell proliferation.	WB, cell viability assays and xenograft models	Ge et al. (99)
Multiple myeloma	OPM-2, 8226, MM1S and HS-Sultan	IRS1 participates in a feedback loop that leads to mTOR inhibitor resistance.	WB	Shi et al. (100)
Hairy cell leukemia	Primary samples	Gain-of-function mutations in IRS1 contribute to resistance to vemurafenib (BRAF^V600E^ inhibitor).	Deep targeted mutational and copy number analysis	Durham et al. (102)

Abbreviations: IP, immunoprecipitation; WB, western blotting; MPN, myeloproliferative neoplasm; MDS, myelodysplastic syndrome; qPCR, quantitative polymerase chain reaction; ALL, acute lymphoblastic leukemia; MC, metaphase cytogenetics; FISH, fluorescence in situ hybridization.
